# Godtfredsen syndrome – recurrent clival chondrosarcoma with 6 years follow up: a case report and literature review

**DOI:** 10.1186/s12883-022-02654-w

**Published:** 2022-04-11

**Authors:** Yong Zheng Wai, Yong Yuin Chong, Noraini Mohd Dusa, Yin Peng Lai, Lik Thai Lim

**Affiliations:** 1 Ophthalmology Department, Kuala Lumpur Hospital, Kuala Lumpur, Malaysia; 2 Ophthalmology Department, Ampang Hospital, Ampang Jaya, Malaysia; 3 Pathology Department, Kuala Lumpur Hospital, Kuala Lumpur, Malaysia; 4 Universiti Malaysia Sarawak (UNIMAS), Kota Samarahan, Sarawak Malaysia

**Keywords:** Godtfredsen syndrome, Clival chondrosarcoma, Clival syndrome

## Abstract

**Background:**

We report a rare case of Godtfredsen syndrome caused by clival chondrosarcoma and perform a review of literatures. This article also explains the clinico-anatomical correlation of this rare neurological syndrome.

**Case presentation:**

A 22-year-old gentleman presented with binocular diplopia. Clinical examination revealed an isolated right abducent nerve and right hypoglossal nerve palsy, with other cranial nerves intact.

Neuroimaging revealed a right clival mass. Supraorbital craniotomy and tumour debulking were done in the same year. Histopathological examination showed low-grade chondrosarcoma.

After 5-years of default, he came back with the tumour enlarged. He underwent a right orbitozygomatic craniotomy and tumour excision with 33 cycles of radiotherapy.

Despite two surgeries and radiotherapy, the abducent nerve and hypoglossal nerve did not improve throughout 6 years of follow-up. Cranial nerve VI palsy is not always a false localizing sign, in Godtfredsen syndrome it serves as a localizing sign.

**Conclusion:**

To the best of our knowledge, this is the first case report of Godtfredsen Syndrome secondary to clival chondrosarcoma. Cranial nerve VI and XII palsy with no involvement of other cranial nerves, most likely the pathology is located at the clivus.

## Background

Godtfredsen syndrome is a rare neurological syndrome that involved abducens nerve (Cranial Nerve VI) and hypoglossal nerve (Cranial Nerve XII) [1]. It spares the cranial nerve in between VI and XII, which often leads the clinician to think of the CN VI palsy as merely a false localizing sign. Our case is the first reported clival chondrosarcoma that gave rise to Godtfredsen syndrome. This article also explains the clinico-anatomical correlation of this rare neurological syndrome.

## Case presentation

A 22-year-old gentleman presented with a one-year history of gradual worsening of horizontal binocular diplopia. The diplopia was worst on his right lateral gaze. He denied any symptoms of raised intracranial pressure or any head trauma. There was no blurring of vision and no visual field loss. He appeared fit and healthy otherwise. No family history of malignancy and no significant psycho-social history.

Neurological examination revealed a right abducens nerve and right hypoglossal nerve palsy, sparing the other cranial nerves. There was no long tract sign or cerebellar sign. Visual acuity was 6/6 in both eyes, and a normal visual field test.

Neuroimaging showed a lesion located at the right spheno-occipital region with epicentre at clivus causing destruction to adjacent bone. He then underwent a right supraorbital craniotomy and debulking of the tumour. Histopathological examination revealed low-grade chondrosarcoma (immunochemical staining profile: S100 positive, D2–40 positive).

Eight months post-operatively, Magnetic Resonant Imaging (MRI) of the brain showed residual mass extending to the cavernous sinus. His right abducens nerve and hypoglossal nerve remains impaired after the surgery. The patient was not keen on any radiotherapy offered by the oncologist and subsequently defaulted for 5 years.

**Fig. 1:**
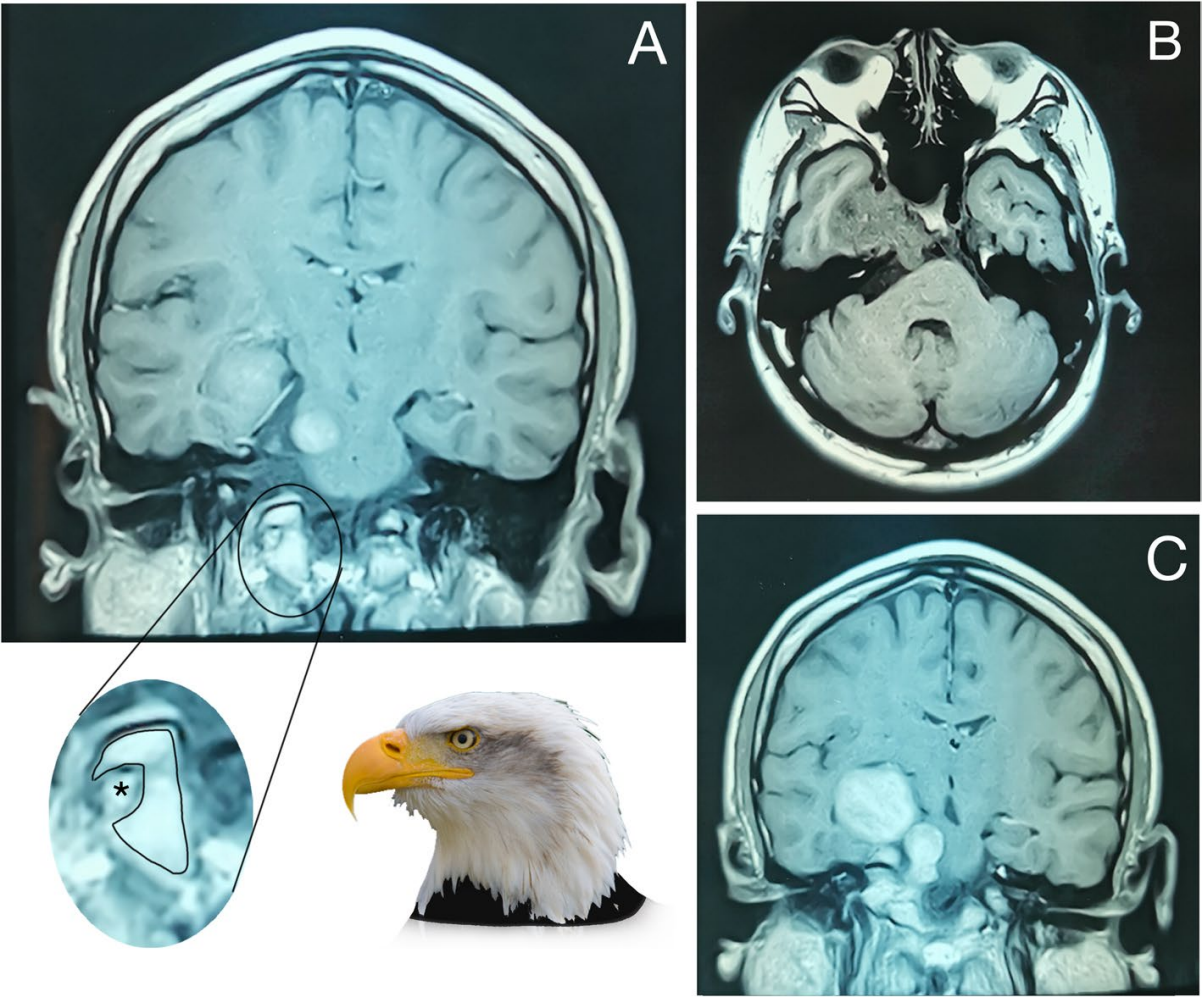
**A** Coronal view of the MRI brain shows residual tumour at the right basiocciput of the clivus. Enlarged photo shows tumour involving the right hypoglossal canal (in asterisk, *) Hypoglossal canal surrounded by jugular tubercle and occipital condyle resembles eagle head. (Eagle head image from Wikimedia Commons). **B** Axial view of MRI brain shows right clival mass at prepontine cistern causing compression the pons. **C** Residual clival chondrosarcoma extending to the right side sella compressing and displacing pituitary gland to the left

**Fig. 2:**
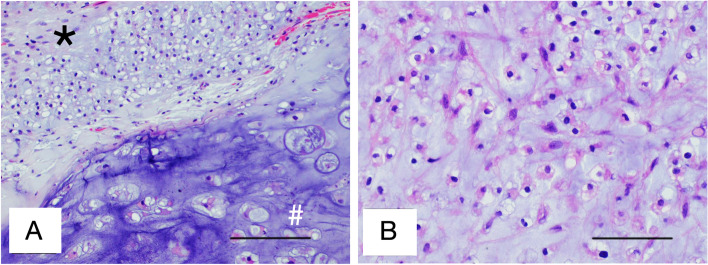
**A** Histopathologic picture of excised tissue with hematoxylin and eosin staining, showing cellular grade 2 area (in asterisk, *) seen in most fragments with area of grade 1 (in hash, #). This slide shows the border between grade 1 and grade 2 area. **B** Grade 2 chondrosarcoma with moderate nuclear pleomorphism set within a myxoid stroma. Scale bars: 100 μm (**A**) and 50 μm (**B**)

When he returned for his follow up, he reported no new symptoms, with his clinical condition status quo. There was no sign of raised intracranial pressure. An MRI revealed that the lesion over the right clivus has enlarged causing the right internal carotid artery displacement and right cavernous sinus compression (Fig. 1). He then underwent a right orbitozygomatic craniotomy with excision of the tumour. However, parts of tumour that had invaded the clivus were left behind. Histopathological examination showed central chondrosarcoma (Grade 2) which is strongly and diffusely immunoreactive to S100 (Fig. 2).

**Fig. 3:**
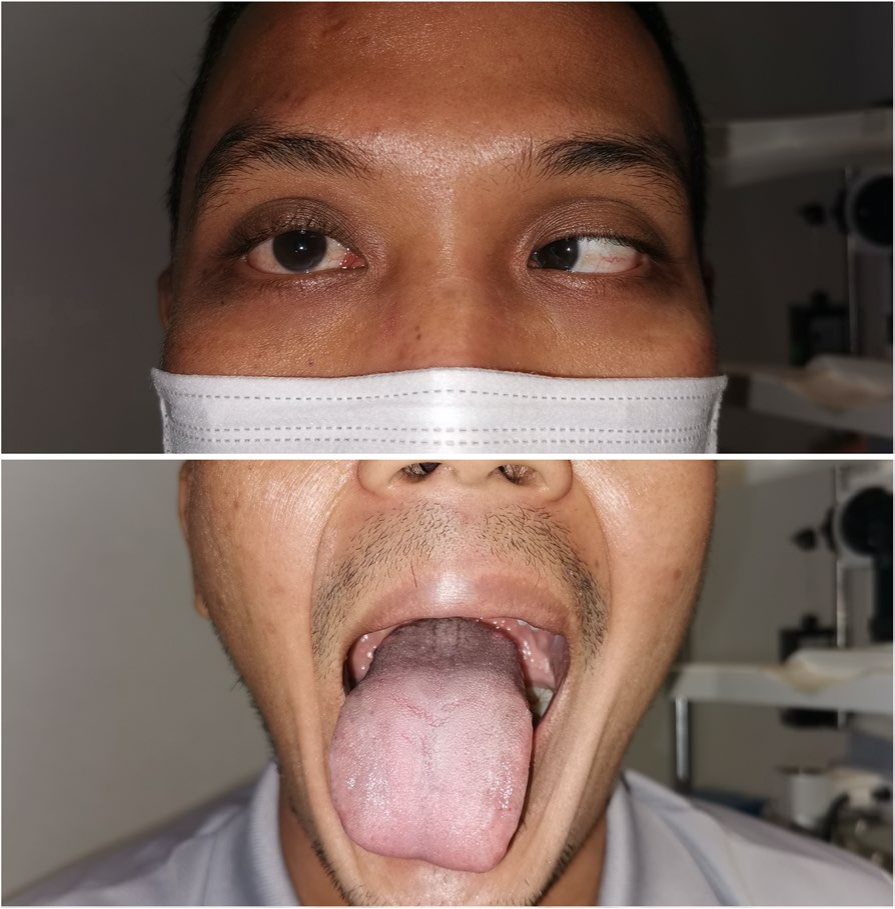
Right eye unable to abduct pass midline on dextroversion. Tongue deviated to the right upon tongue protrusion. Right abducent nerve (cranial nerve VI) and right hypoglossal nerve palsy (cranial nerve XII)

He has completed radiotherapy of 33 cycles over a period of 6 months after the surgery. On the latest Ophthalmology clinic visit, the VI and XII palsies still persist (Fig. 3). Other neurological examination was normal and he has no new complaints. During the latest visit, neurosurgical team has no active surgical intervention. He is now under neurosurgical and ophthalmology follow up regularly.

## Discussion and conclusions

Godtfredson syndrome is a rare neurological manifestation comprised of concomitant abducens nerve (Cranial Nerve VI) and hypoglossal nerve (Cranial Nerve XII) was first described by Erik Godtfredsen relating nasopharyngeal carcinoma [1]. The presence of these signs narrow down the location of pathology to the clivus [2].

**Fig. 4:**
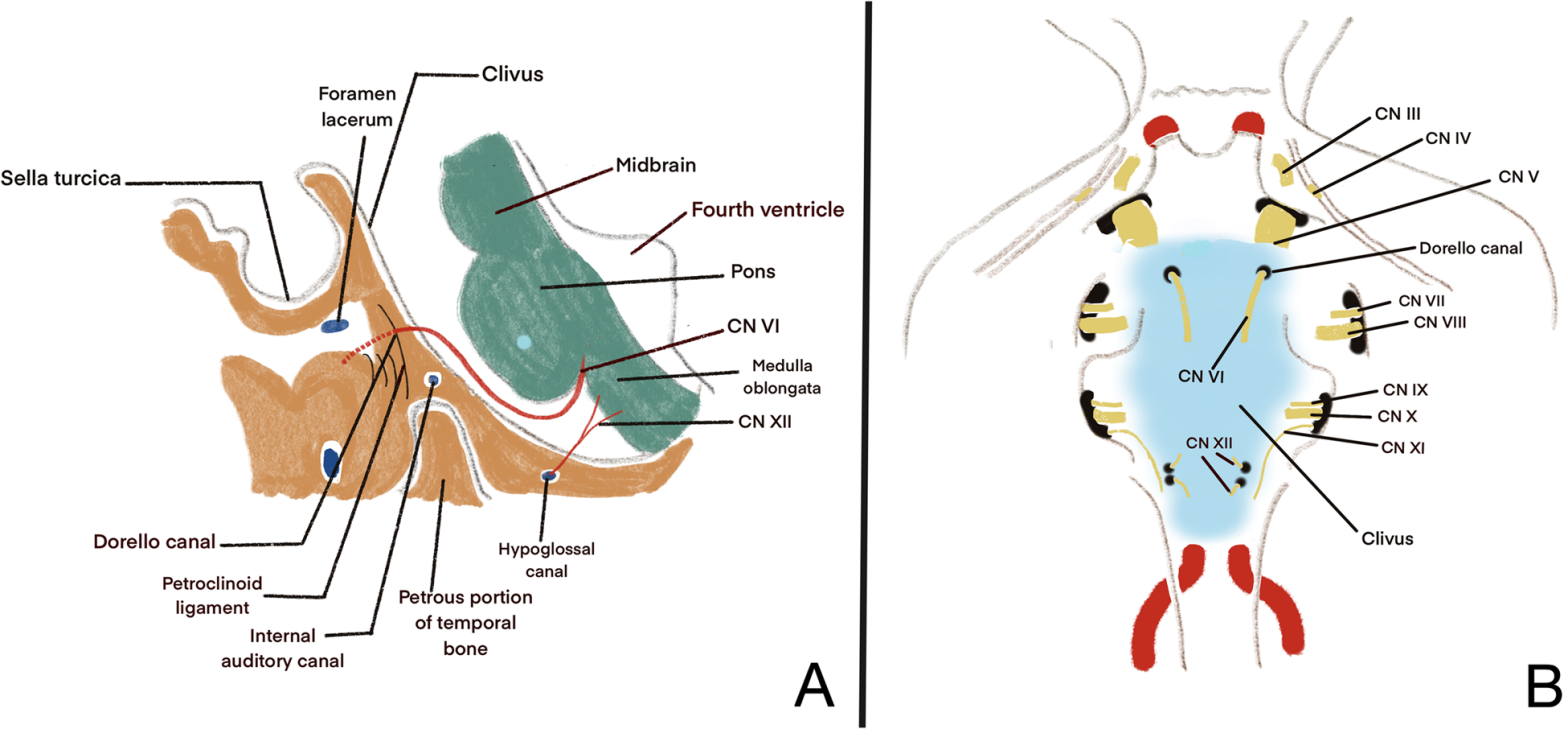
**A** Illustration of the sagittal section of the clivus, shows CN VI and XII in relation to the clivus. **B** Posterior view of the clivus shows CN VI and XII are located more medially compared to other cranial nerves on the clivus

The abducens nerve exits the pons at the pontomedullary sulcus to the dural foramen, being the cisternal segment of cranial nerve VI [3].This portion of abducens nerve lie superior to the cisternal segment of hypoglossal nerve that exits at the lower two-thirds of the olive in preolivary sulcus and enter hypoglossal canal [4]. Both of the structures lies on the medial portion of clivus compared to cranial nerve V, VII, VIII, IX and X (Fig. 4). Thus, midline lesion located at clivus can disrupt cranial nerve VI and XII without involving other cranial nerves.

Isolated cranial nerve VI and XII palsies are rare. A literature review found 9 cases in the past 20 years (Table 1). 55.5% (5/9) of the cases are secondary to metastatic diseases at clivus [2, 5–8]. Amongst the 9 cases, 66.7% (6/9) are tumour origin. Only one case is caused by a tumour arisen from clivus – clival chondroma. Clival chondrosarcoma commonly involved multiple contiguous cranial nerves unlike chondroma [9]. We hereby report the first clival chondrosarcoma case associated with Godtfredsen syndrome.

**Table 1:** Literatures review of Godtfredsen Syndrome

Author, year	No of patient	Sex, Age	Pathology
Keane, 2000 [2]	5		-Clivus chondroma -Prostate metastasis -Pancreas metastasis -Ovary metastasis -Self-limiting cranial polyneuropathy
Alessi, 2003 [5]	1	F/66	Leiomyoma metastasis
Lekhjung, 2011 [8]	1	M/27	Rectal adenocarcinoma metastasis
Jacob, 2015 [6]	1	F/2	Anaerobic mastoiditis
Amalnath, 2018 [7]	1	F/30	Retroclival subdural hematoma

Cranial nerve VI palsy is not always a false localising sign. Due to the medially located cranial nerves VI and XII, clivus lesion can directly affect the cisternal segment of cranial nerve VI and XII. In such a scenario, cranial nerve VI palsy will becomes a localising sign.

In the case of isolated cranial nerve VI and XII palsies, this should alarm the clinician of the possibility of a more sinister pathology at the clivus.

## Data Availability

All data and materials gathered during this study are included in this study.

## Abbreviations

MRI: Magnetic Resonance Imaging
CN: Cranial Nerve
